# Draft genome sequence of *Exiguobacterium* sp. strain HSB13, a thermophilic bacterium isolated from a terrestrial hot spring in Algeria

**DOI:** 10.1128/mra.00463-26

**Published:** 2026-10-08

**Authors:** Khelifa Bouacem, Chahira Zerouki, Nitin Bayal, Suvi Kuittinen, Shubha Rani Sharma, Ari Pappinen, Ossi Turunen

**Affiliations:** 1 Department of Biochemistry and Microbiology, Faculty of Biological and Agricultural Sciences (FBAS), University Mouloud Mammeri of Tizi-Ouzou (UMMTO) 124198, Tizi Ouzou, Algeria; 2 Laboratory of Cellular and Molecular Biology (LCMB), Microbiology Team, Faculty of Biological Sciences (FBS), University of Sciences and Technology of Houari Boumediene (USTHB) 61758, Algiers, Algeria; 3 School of Forest Sciences, University of Eastern Finland 163043, Joensuu, Finland; 4 Department of Biological and Environmental Sciences, University of Eastern Finland 163043, Joensuu, Finland; 5 Department of Computing, University of Turku 8058, Turku, Finland; 6 Department of Bioengineering and Biotechnology, Birla Institute of Technology 28698 , Ranchi, Jharkhand, India; Indiana University Bloomington, Bloomington, Indiana, USA

**Keywords:** biofilm, *Exiguobacterium *sp. strain HSB13, enzymes, exopolysaccharides, whole-genome sequence

## Abstract

*Exiguobacterium* sp. HSB13 is a moderately thermophilic bacterium isolated from an Algerian hot spring. Its genome comprises 3,238,983 bp assembled into 62 contigs and encodes 3,284 protein-coding genes, with a GC content of 47.06%. Genome analysis suggests a genomic potential for exopolysaccharide biosynthesis and biofilm formation.

## ANNOUNCEMENT

*Exiguobacterium* species exhibit a versatile metabolism and strong stress-response systems. These traits make them promising candidates for several biotechnological applications (1–7).

Water samples were collected from Assif El Hammam hot spring, Bejaia, Algeria (36°43′51″ North, 4°34′56’’ East). Enrichment cultures and isolation were performed using medium growth containing (g/L): glucose (3.6); NH_4_Cl (1); K_2_HPO_4_ (0.3); KH_2_PO_4_ (0.3); NaCl (1.0); KCl (0.1); CaCl_2_·2H_2_O (0.1); MgCl_2_·6H_2_O (0.25); yeast extract (1.0); and biotrypcase (2.0). The pH was adjusted to 7.0. The plates were inoculated and incubated in aerobic conditions at 50°C for 24–72 h (8). A single colony was selected and purified. The isolate HSB13 was then characterized using Gram staining and microscopic examination.

Genomic DNA was extracted using the Nanobind PanDNA Kit (Pacific Biosciences, USA) following the manufacturer’s instructions, with an initial lysozyme treatment to facilitate bacterial cell lysis. The bacterial cells were resuspended in phosphate-buffered saline (PBS), and 20 µL of the resulting cell suspension was used as input for DNA extraction. Genomic DNA libraries were prepared using the Illumina DNA Prep Kit (Illumina, USA). A total of 280 ng of genomic DNA was used for library preparation with BIDGEN Nextera-compatible indexing primers. Libraries were amplified for seven PCR cycles and purified using Illumina sample purification beads (SPB). The library pool was sequenced on an Illumina MiSeq platform using a MiSeq v3 600-cycle flow cell at a final library concentration of 9.5 pM. The sequencing run was manually configured before sequencing with 326 cycles for Read 1 and 278 cycles for Read 2 based on the higher sequencing quality of Read 1 compared with Read 2. The run generated paired-end reads of 326 (Read 1) and 278 bp (Read 2) with a target output of approximately 1–2 million paired-end reads per sample. Illumina paired-end reads were processed with fastp v1.0.1 (9) for adapter trimming and quality filtering using default parameters. A total of paired-end 9,086,758 reads were obtained, and 9,008,724 (99.14%) were retained after quality control. The filtered data set comprised 2,009,396,119 bases, corresponding to 620× coverage of the 3,238,983 bp genome. Quality filtering improved the Q30 score from 76.8 to 90.0%, and reads with low-quality scores, excessive ambiguous bases (N), or insufficient length were discarded. Genome assembly was performed using SPAdes v4.0.0 (10) in isolate mode with paired-end reads (orientation: fr). The assembly was run with eight threads using automatic k-mer selection and repeat resolution enabled. Coverage cutoff and mismatch correction were disabled. Genome annotation was conducted using National Center for Biotechnology Information (NCBI) Prokaryotic Genome Annotation Pipeline (PGAP) (11) and RAST subsystem (12). No plasmid-associated sequences were detected with PlasmidFinder v2.1.6 (13), and no CRISPR arrays were found using CRISPRFinder (CRISPR-Cas++ v2.0.1) (14) (Table 1).

**TABLE 1:** Draft genome features of *Exiguobacterium* sp. strain HSB13 generated by the NCBI Prokaryotic Genome Annotation Pipeline (PGAP)

Features	Values
Draft genome size	3,238,983 bp
Number of contigs	62
Number of scaffolds	62
GC content	47.06%
Contig L50	1
Contig N50	2,251,873 bp
Genes (total)	3,412
CDSs (total)	3,303
Genes (coding)	3,284
Genes (RNA)	109
rRNAs (5S, 16S, 23S)^*a*^	11, 10, 17
tRNAs	67
ncRNAs	4
Pseudogenes (total)	19
CRISPR arrays	None found
Plasmids	None found

^*a*^ rRNA counts include complete and partial gene predictions identified by the PGAP.

HSB13 is gram-positive, short rod-shaped, motile, and non-sporulating. The strain was orange-pigmented and facultative anaerobe. The BLAST+ blastn (Galaxy v2.16.0+) (15) of the assembled draft genome indicated *Exiguobacterium* sp. ZWU0009 (CP018057.1) as the closest reference genome, sharing 97.83% identity. Genome completeness was estimated at 99.4% using CheckM (v1.2.5) (16) calculated on the PGAP gene set with the *Exiguobacterium* CheckM marker set. The RAST subsystem annotation revealed that the *Exiguobacterium* sp. HSB13 draft genome encodes a complete exopolysaccharide biosynthesis cluster, suggesting a potential genetic basis for EPS production and biofilm formation (17).

## Data Availability

This draft genome has been deposited at DDBJ/ENA/GenBank under the accession JBUZOM000000000. The version described in this paper is version JBUZOM010000000. The raw sequencing data have been deposited in the NCBI Sequence Read Archive (SRA) under BioProject accession PRJNA1422484, including run accession SRR38225775.
